# Long-Term Adverse Effect of Liver Stiffness on Glycaemic Control in Type 2 Diabetic Patients with Nonalcoholic Fatty Liver Disease: A Pilot Study

**DOI:** 10.3390/ijms232012481

**Published:** 2022-10-18

**Authors:** Alessandro Mantovani, Antonio Taverna, Davide Cappelli, Giorgia Beatrice, Alessandro Csermely, Elena Sani, Christopher D. Byrne, Giovanni Targher

**Affiliations:** 1 Section of Endocrinology, Diabetes and Metabolism, Department of Medicine, University of Verona, 37126 Verona, Italy; 2 Nutrition and Metabolism, Faculty of Medicine, University of Southampton, Southampton SO17 1BJ, UK; 3 Southampton National Institute for Health and Care Research Biomedical Research Centre, and University Hospital Southampton, Southampton General Hospital, Tremona Road, Southampton SO16 6YD, UK

**Keywords:** nonalcoholic fatty liver disease, NAFLD, MAFLD, metabolic associated fatty liver disease, type 2 diabetes, T2DM, fibrosis, liver stiffness

## Abstract

Currently, there are limited data regarding the long-term effect of liver stiffness on glycaemic control in patients with type 2 diabetes mellitus (T2DM) and nonalcoholic fatty liver disease (NAFLD). We prospectively followed an outpatient sample of 61 consecutive postmenopausal women with T2DM and NAFLD who had baseline data on liver ultrasonography and Fibroscan^®^-assessed liver stiffness measurement (LSM) in 2017 and who underwent follow-up in 2022. Haemoglobin A1c (HbA1c) was measured both at baseline and follow-up. At baseline, 52 patients had NAFLD (hepatic steatosis) alone, and 9 had NAFLD with coexisting clinically significant fibrosis (defined as LSM ≥ 7 kPa on Fibroscan^®^). At follow-up, 16 patients had a worsening of glycaemic control (arbitrarily defined as HbA1c increase ≥ 0.5% from baseline). The prevalence of NAFLD and coexisting clinically significant fibrosis at baseline was at least three times greater among patients who developed worse glycaemic control at follow-up, compared with those who did not (31.3% vs. 8.9%; *p* = 0.030). In logistic regression analysis, the presence of NAFLD and clinically significant fibrosis was associated with an approximately 4.5-fold increased likelihood of developing worse glycaemic control at follow-up (odds ratio 4.66, 95% confidence interval 1.07–20.3; *p* = 0.041), even after adjustment for baseline confounding factors, such as age, body mass index, haemoglobin A1c (or HOMA-estimated insulin resistance) and use of some glucose-lowering agents that may positively affect NAFLD and liver fibrosis. In conclusion, our results suggest that the presence of Fibroscan^®^-assessed significant fibrosis was associated with a higher risk of developing worse glycaemic control in postmenopausal women with T2DM and NAFLD.

## 1. Introduction

Nonalcoholic fatty liver disease (NAFLD) has become the most common cause of chronic liver diseases worldwide, affecting up to ~30% of adults in the general population [1]. NAFLD also affects up to ~70% of patients with type 2 diabetes mellitus [T2DM] and almost all patients with severe obesity [2]. Worryingly, the global prevalence of NAFLD is expected to increase dramatically in the near future, in parallel with the increasing rates of obesity and T2DM globally [1]. T2DM and NAFLD represent a “vicious circle”, whereby the presence of one condition adversely affects the other and vice versa [3]. Compared with subjects without T2DM, patients with T2DM are more likely to have or develop the more advanced forms of NAFLD, such as nonalcoholic steatohepatitis (NASH), advanced fibrosis or cirrhosis [4,5]. In this context, a recent systematic review reported that the global prevalence of biopsy-confirmed NASH among patients with T2DM is nearly 40% and that the global prevalence of advanced fibrosis in this patient population is around 15–20% [2]. On the other side of this “vicious circle”, NAFLD may precede and/or promote the development of T2DM, possibly via worsening systemic/hepatic insulin resistance and dysregulated production of several hepatokines and proinflammatory cytokines [4,6]. An updated meta-analysis of 33 observational cohort studies (including about 500,000 individuals) showed that NAFLD was significantly associated with a ~2.2-fold increased risk of new-onset T2DM over a median period of 5 years. This risk paralleled the underlying severity of NAFLD (especially higher stage of liver fibrosis) [7].

On this background of evidence, in 2022, the American Diabetes Association (ADA) guidelines recommended that individuals with T2DM and elevated serum liver enzyme levels or NAFLD on ultrasonography should be evaluated for the presence of liver fibrosis [8]. This also presupposes the need to better assess, on the one hand, how long-term glycaemic control may affect the development and progression of NAFLD and, on the other hand, how NAFLD and its more advanced forms may affect long-term glycaemic control in patients with T2DM. While convincing evidence indicates that suboptimal glycaemic control may predispose a patient to the development of more advanced forms of NAFLD [9,10,11,12,13,14,15,16], to date there is little information regarding the long-term effect of NAFLD with increasing levels of liver fibrosis on glycaemic control in patients with T2DM. Recognition of a possible long-term adverse effect of NAFLD with coexisting liver fibrosis on glycaemic control might have important clinical implications, as it may further reinforce the need for a multidisciplinary, patient-centred approach to patients with T2DM and advanced NAFLD, as well as the need for a tailored pharmacotherapy in this patient population (preferring the use of glucose-lowering agents with potential hepatoprotective effects), in order to achieve better glycaemic control and prevent future NAFLD-related hepatic and extra-hepatic complications.

Thus, the main aim of this observational longitudinal pilot study was to examine whether T2DM patients with NAFLD and coexisting clinically significant fibrosis (as noninvasively assessed by liver ultrasonography and vibration-controlled transient elastography (VCTE)) had a worsening of glycaemic control over time, compared with their counterparts with NAFLD alone.

## 2. Results

Of the 61 postmenopausal women with T2DM included in the study, 52 (85%) patients had NAFLD (hepatic steatosis) alone, and 9 (15%) patients had NAFLD and coexisting clinically significant fibrosis (i.e., defined as liver stiffness measurement (LSM) ≥ 7 kPa on Fibroscan^®^) at baseline; 7 of these 9 patients with NAFLD and coexisting significant fibrosis had a LSM ≥ 8.2 kPa (which is another more stringent cut-off used for defining the presence of clinically significant fibrosis).

Table 1 shows the main clinical and biochemical characteristics of the study participants at baseline (year 2017) who were stratified by worsening of glycaemic control at follow-up (year 2022). Baseline LSM values on Fibroscan^®^ were significantly higher in patients who developed worse glycaemic control at follow-up compared with those who did not (median LSM: 6.6 (IQR 5.4–8.6) vs. 4.4 (3.6–5.6) kPa; *p* = 0.005). Similarly, the proportion of patients with NAFLD and clinically significant fibrosis (i.e., LSM ≥ 7 kPa) at baseline was greater among those who developed worse glycaemic control at follow-up compared with those who did not (31.3% vs. 8.9%; *p* = 0.030). Again, the proportion of those with NAFLD and LSM ≥ 8.2 kPa at baseline was greater among those who developed worse glycaemic control at follow-up compared with those who did not (25.0% vs. 6.7%; *p* = 0.044). Conversely, the two patient groups did not significantly differ for other clinical and biochemical characteristics at baseline, such as age; BMI; smoking history; blood pressure; HbA1c; proportion of those with HbA1c from 7% to 8% or those with HbA1c > 8%; plasma lipid profile; HOMA-IR score; serum liver enzymes; kidney function parameters; prevalence of ischaemic heart disease or stroke; and use of glucose-lowering, antihypertensive, lipid-lowering or antiplatelet agents.

Table 2 summarizes the main clinical and biochemical characteristics of the study participants at follow-up, who were stratified by severity of NAFLD at baseline. Compared with those with NAFLD alone, patients with NAFLD and clinically significant fibrosis had markedly higher levels of HbA1c at follow-up (HbA1c 8.4 ± 2.1% vs. 6.9 ± 0.9%; *p* < 0.001). Moreover, the proportion of those with HbA1c >8% at follow-up was also significantly greater in patients with NAFLD and clinically significant fibrosis than in those with NAFLD alone (55.6% vs. 11.1%; *p* = 0.003). All other clinical and biochemical characteristics recorded at follow-up were not significantly different between the two groups of patients, including the use of glucose-lowering, antihypertensive, lipid-lowering or antiplatelet agents.

Table 3 shows the association between the severity of NAFLD at baseline and worsening of glycaemic control at follow-up (arbitrarily defined as HbA1c increase ≥ 0.5% from baseline). The presence of NAFLD and significant fibrosis was significantly associated with an approximately 4.5-fold increased risk of worsening of glycaemic control at follow-up (unadjusted-OR 4.66, 95% CI 1.07–20.3; *p* = 0.041). The adjustment for age and BMI (model 1) or for the baseline use of some specific glucose-lowering agents, such as glucagon-like peptide 1 (GLP-1) receptor agonists (model 2), sodium-glucose cotransporter-2 (SGLT2) inhibitors (model 3) or pioglitazone (model 4) that might favourably affect NAFLD and liver fibrosis did not weaken the strength of this association. Almost identical results were found even when we used LSM ≥ 8.2 kPa instead of ≥7 kPa (by excluding two patients from the analysis) for defining the presence of clinically significant fibrosis Further adjustment for baseline HbA1c levels did not change the strength of the association between the severity of NAFLD at baseline and risk of developing worsening of glycaemic control at follow-up (Supplementary Table S1). Almost identical results were also observed when we included the HOMA-IR score (instead of HbA1c) as the covariate in these multivariable logistic regression models.

In Table 4 are reported the associations between the severity of NAFLD at baseline and four increasing categories of worsening of glycaemic control at follow-up (arbitrarily defined as HbA1c increases ≤ 0.19%, from 0.20% to 0.49%, from 0.50% to 0.99%, and ≥ 1%, respectively). The results of these ordered logistic regression models were superimposable upon those reported in Table 3, showing that the presence of NAFLD and clinically significant fibrosis at baseline was strongly associated with an increased risk of worsening of glycaemic control at follow-up, even after adjustment for potential confounding factors. This association remained significant even when we further adjusted the data for baseline HbA1c levels (Supplementary Table S2). Almost identical results were also observed when we included the HOMA-IR score (instead of HbA1c) as the covariate in these ordered logistic regression models.

## 3. Discussion

The main findings of our longitudinal pilot study involving postmenopausal women with T2DM and NAFLD are as follows: (a) compared with NAFLD alone, the presence of NAFLD and clinically significant fibrosis at baseline (as noninvasively assessed by liver ultrasound and VCTE examinations) was significantly associated with an approximately 4.5-fold increased risk of glycaemic worsening at follow-up (5 years later); and (b) this significant association persisted even after adjusting for baseline confounding factors such as age, BMI, HbA1c (or HOMA-IR score) and use of some glucose-lowering agents (GLP-1 receptor agonists, SGLT-2 inhibitors, or pioglitazone) that may positively affect hepatic steatosis and fibrosis. In this study, we used an increase in HbA1c of at least 0.5% from baseline to define glycaemic worsening. This HbA1c increase has been used as a marker of worsening of glycaemic control in patients with T2DM in other published studies [17,18].

In 2022, the ADA scientific guidelines recommended that patients with T2DM and elevated serum liver enzymes or NAFLD on ultrasonography should be evaluated for presence of liver fibrosis [8], thereby supporting the need for a better understanding of how long-term glycaemic control may impact on the risk of NAFLD and, on the other hand, how NAFLD may impact on long-term glycaemic control in T2DM.

To date, there is evidence showing that poor glycaemic control is associated with a higher likelihood of having NASH or advanced fibrosis [4,10]. For instance, in a cross-sectional study of 713 patients with biopsy-confirmed NAFLD (~50% of whom had established T2DM), Angelopoulos et al. reported that patients with poor glycaemic control were more likely to have NASH and advanced fibrosis compared with those with good glycaemic control [19]. In a small study involving 39 patients with biopsy-proven NAFLD who were followed for a median period of 2.4 years, Hamaguchi et al. showed that insulin use and lower HbA1c levels were associated with a significant improvement in liver fibrosis, independent of age, sex and BMI [20]. In a cross-sectional study of nearly 1900 individuals with ultrasound-detected NAFLD, Tanaka et al. reported that a HbA1c level ≥6.5% (≥48 mmol/mol) was associated with greater severity of liver fibrosis, as assessed noninvasively by fibrosis (FIB)-4 index [21].

Little information is available to date about the long-term effects of NAFLD and its more advanced forms on long-term glycaemic control in people with T2DM. In this context, in a small cross-sectional study of 230 individuals who underwent Fibroscan^®^, Patel et al. showed that patients with NAFLD were more likely to have HbA1c levels ≥7% (≥53 mmol/mol) and to be treated with insulin [13]. Preliminary cross-sectional evidence has also shown that liver fat content (as assessed by proton spectroscopy) may be the principal factor explaining the daily amount of insulin required to achieve good glycaemic control in patients with insulin-treated T2DM [11].

Collectively, therefore, the results of our study corroborate and expand the aforementioned findings, showing that the baseline presence of NAFLD and coexisting significant fibrosis (as assessed by ultrasonography and VCTE) were strongly associated with a worsening of glycaemic control at follow-up, irrespective of age, BMI, HbA1c, HOMA-IR score and baseline use of certain glucose-lowering agents.

The most obvious explanation for our findings is that the association between advanced NAFLD and worsening of glycaemic control at follow-up might arise from shared metabolic risk factors. However, it is important to note that in our study, the association of NAFLD and clinically significant fibrosis with worsening of glycaemic control remained statistically significant even after adjusting for some important confounding factors at baseline, including the use of GLP-1 receptor agonists, SGLT-2 inhibitors or pioglitazone that may positively affect NAFLD (steatosis) and liver fibrosis. Notably, as reported in Table 1 and Table 2, the use of these and other glucose-lowering agents, both at baseline and at follow-up, did not significantly differ between patients with NAFLD alone and those who had NAFLD and coexisting clinically significant fibrosis at baseline. Hence, on the basis of the results of our study, it is also possible to hypothesize that the presence of NAFLD and coexisting clinically significant fibrosis might partly contribute to glycaemic worsening, possibly through exacerbation of systemic and hepatic insulin resistance, and increased production of multiple hepatokines (such as, for example, fetuin A, fetuin B or fibroblast growth factor-21) and proinflammatory cytokines (such as, for example, tumour necrosis factor-α or interleukin-6) [4,6,9].

Although further research is certainly needed, our findings may have some important clinical implications, as they further support the need for a multidisciplinary and holistic approach to patients with T2DM and advanced NAFLD, as well as the need for a tailored drug treatment of NAFLD in this specific patient population [22,23,24]. In particular, although there are no licensed treatments for NAFLD, three different classes of glucose-lowering drugs (peroxisome proliferator-activated receptor agonists, GLP-1 receptor agonists and SGLT-2 inhibitors) showed promise in the treatment of this common liver disease. Specifically, pioglitazone and GLP-1 receptor agonists (mostly subcutaneous liraglutide and semaglutide) improved individual histological features of NASH or achieved histological resolution of NASH without worsening of fibrosis. SGLT-2 inhibitors (mostly dapagliflozin and empagliflozin) improved plasma aminotransferase levels and liver fat content, as assessed by magnetic resonance-based techniques [25,26,27,28]. A recent consensus report by the ADA and the European Association for the Study of Diabetes on management of T2DM also suggested for the first time that individuals with T2DM at an intermediate to high risk of liver fibrosis should be considered for treatment with pioglitazone and/or a GLP-1 receptor agonist with evidence of benefit [29].

Our study has some important limitations that should be mentioned. First, the observational design of the study precludes making any causal inferences. Second, the number of participants was small, and the study included only Caucasian postmenopausal women with T2DM and NAFLD. Hence, our results cannot be necessarily generalizable to other patient groups, including, for example, men with T2DM (the investigation of possible sex-related differences is now becoming a priority in NAFLD research [30]). Third, we used only two HbA1c measurements for each study participant, one performed in 2017 (at baseline) and one performed in 2022 (at follow-up). Hence, the lack of repeat measurements of HbA1c between 2017 and 2022 does not allow us to have detailed information about the temporal trends of HbA1c levels. Fourth, although the further adjustment for HOMA-score did not attenuate the significant association we observed between the presence of NAFLD and clinically significant fibrosis at baseline and the risk of worsening of glycaemic control at follow-up, larger prospective studies are needed to better elucidate the long-term effect of insulin resistance on glycaemic control in patients with T2DM and advanced NAFLD. In addition, due to the small sample size of the study, it is important to note that time-varying covariates, such as changes in glucose-lowering agents over the follow-up, cannot be included in multivariable logistic regression models. Fifth, we did not perform a liver biopsy or magnetic resonance elastography for staging liver fibrosis at baseline nor a VCTE examination at follow-up. Hence, the possible differential effects of hepatic steatosis, inflammation, ballooning and fibrosis on glycaemic worsening over time cannot be accurately assessed in our study. However, both ultrasonography and VCTE (Fibroscan^®^) are two noninvasive methods that are widely used for diagnosing and staging NAFLD in clinical practice [31], although ultrasonography is characterized by interobserver and intraobserver variability [32,33] and Fibroscan^®^-assessed LSMs may be affected not only by hepatic fibrosis but also by severe hepatic steatosis and inflammation [34]. Finally, we cannot definitely exclude the possibility that other unmeasured factors might partly explain the observed associations.

Despite these limitations, our study has some important strengths, including the consecutive enrolment of the study population and the completeness of our database. Additionally, both liver ultrasound and VCTE examinations were performed by a single expert physician, who was blinded to participants’ clinical and biochemical details, thereby eliminating possible assessment bias and interobserver variability. However, we cannot exclude a certain degree of intraobserver variability in the diagnosis of hepatic steatosis on ultrasonography [32,33]. Finally, we excluded T2DM patients with important comorbidities (e.g., cirrhosis, cancer and end-stage kidney disease), as we believe that the inclusion of patients with such comorbidities might have confounded the interpretation of data.

In conclusion, the results of our longitudinal pilot study suggest that the presence of NAFLD and clinically significant fibrosis at baseline was associated with a markedly higher risk of worsening of glycaemic control at follow-up (5 years later) in postmenopausal women with T2DM and NAFLD. The strength of this association was not weakened by adjustment for important baseline confounding factors such as age, BMI, HbA1c, HOMA-IR score and use of some specific glucose-lowering agents (such as pioglitazone, GLP-1 receptor agonists or SGLT-2 inhibitors) that might favourably affect NAFLD and liver fibrosis. Further studies are certainly needed to confirm these data in other patient cohorts and to better understand whether the prescription of certain glucose-lowering drugs with potential hepatoprotective effects may increase the probability of achieving good glycaemic control in patients with T2DM and advanced NAFLD.

## 4. Methods and Materials

### 4.1. Patients

We studied 61 Caucasian postmenopausal women with T2DM and NAFLD consecutively attending our diabetes outpatient service who had data on liver ultrasonography and VCTE that were performed in the year 2017 (baseline) and who subsequently underwent a diabetic visit in the first 6 months of 2022 (follow-up). The exclusion criteria of the study were as follows: (a) history of significant alcohol consumption (defined as >20 g of alcohol per day) and other competing causes of hepatic steatosis (e.g., virus, drugs, autoimmunity or hemochromatosis); (b) cirrhosis, cancer and end-stage kidney disease; and (c) chronic use of potentially hepatotoxic drugs. Considering the technical limitations of VCTE methodology, patients with congestive heart failure or free abdominal fluid were also excluded from the study. Most patients enrolled in this study have also been included in our previous studies [5,35].

The local Ethics Committee approved the study protocol. All patients gave their written informed consent for participation in this research.

### 4.2. Clinical and Laboratory Data

Body mass index (BMI) was measured as kilograms divided by the square of height in meters. Blood pressure was measured with a standard sphygmomanometer after the patient had been seated quietly for at least 5 min. Patients were considered to have hypertension if their blood pressure was ≥140/90 mmHg or if they were taking any antihypertensive drugs.

Venous blood samples were collected in the morning after an overnight fast. Complete blood count, glucose, lipids, creatinine, liver enzymes and other biochemical blood parameters were measured using standard laboratory procedures (Roche Cobas 8000; Roche Diagnostics, Basel, Switzerland) at the central laboratory of our hospital. Haemoglobin A1c (HbA1c) was measured using the high-performance liquid chromatography analyser Tosoh-G7 (Tosoh Bioscience Inc., Tokyo, Japan). The Homeostasis model assessment—insulin resistance (HOMA-IR) score was used for estimating insulin resistance. Glomerular filtration rate (GFR) was estimated using the Chronic Kidney Disease Epidemiology Collaboration (CKD-EPI) study equation [36].

Presence of ischaemic heart disease was defined as a documented history of myocardial infarction, angina or coronary revascularizations. Presence of ischaemic stroke was based on medical history and examination and was confirmed by reviewing hospital medical records of patients, including radiology imaging results. Presence of diabetic retinopathy, diagnosed with fundoscopy after pupillary dilation, was also recorded in all patients. All these data were collected both at baseline and at follow-up, except for the HOMA-IR score, which was available only at baseline.

### 4.3. Liver Ultrasonography and VCTE

A single expert physician, who was blinded to participants’ clinical and biochemical details, performed both liver ultrasonography and VCTE examinations at baseline. Hepatic steatosis was diagnosed by using ultrasonography (using an Esaote MyLab 70 ultrasound with a 4 MHz probe, Esaote Group, Genova, Italy), according to specific ultrasonographic characteristics, such as diffuse hyperechogenicity of the liver relative to kidneys, ultrasound beam attenuation and poor visualization of intrahepatic vessel borders and the diaphragm [5,35]. Semiquantitative ultrasonographic indices of hepatic steatosis were not available in this study.

Liver stiffness measurement (LSM) was performed by VCTE using Fibroscan^®^ (Echosens, Paris, France) and an M probe [5,35]. We did not have the Fibroscan^®^ XL probe for patients with severe obesity. The accuracy of the Fibroscan^®^ M probe to identify significant liver fibrosis is excellent in those with overweight or grade 1 obesity (BMI ≤ 35 kg/m^2^). In our study, only four patients had a BMI > 35 kg/m^2^. Our Fibroscan^®^ system was not equipped with the controlled attenuation parameter (CAP) technology for measuring hepatic steatosis [5,35]. LSMs were performed in each patient after at least eight hours of fasting and in the same day of the liver ultrasound examination [5,35]. Further details of the technical background and examination procedures have been described elsewhere [31]. Briefly, each patient’s LSM was considered adequate if it included at least 10 valid measurements, with a success rate > 60% and measurement variability < 30% of the median [5,35]. The presence of clinically significant hepatic fibrosis was defined by the presence of LSM ≥ 7 kPa (which corresponds to Kleiner’s stage F ≥ 2 fibrosis on liver histology) [5,37].

### 4.4. Statistical Analysis

Given the exploratory design of the study, we did not perform an *a priori* sample size calculation. Continuous variables were expressed as means ± SD or medians and inter-quartile ranges (IQR) when indicated, while categorical variables were expressed as proportions. The Fischer’s exact test for categorical variables, the unpaired Student’s *t* test for normally distributed continuous variables and the Mann–Whitney test for non-normally distributed continuous variables (i.e., diabetes duration, plasma triglycerides, HOMA-IR score and Fibroscan^®^-assessed LSM) were used to examine the intergroup differences in main clinical and biochemical characteristics of the study participants, who were stratified either by severity of NAFLD at baseline (NAFLD alone vs. NAFLD and coexisting significant fibrosis) or by an overall worsening of glycaemic control at follow-up (arbitrarily defined as HbA1c increase ≥ 0.5% from baseline).

We tested the independent association between the severity of NAFLD at baseline and worsening of glycaemic control at follow-up (i.e., HbA1c increase ≥ 0.5% from baseline) by using logistic regression analyses. We performed four adjusted logistic regression models. Model 1 was adjusted for age and BMI at baseline; model 2 was adjusted for age, BMI and baseline use of GLP-1 receptor agonists; model 3 was adjusted for age, BMI and baseline use of SGLT2 inhibitors; and, finally, model 4 was adjusted for age, BMI and baseline use of pioglitazone. We also repeated the same multivariable logistic regression models after further adjustment for HbA1c or HOMA-IR score at baseline. Additionally, we performed an ordered logistic regression analysis (also called the ordered logit model, which is a subtype of logistic regression where the Y-category is categorical and ordered) using four increasing categories of worsening of glycaemic control (arbitrarily defined as HbA1c increases at follow-up ≤ 0.19%, from 0.20% to 0.49%, from 0.50% to 0.99%, and ≥1%, respectively) that was included as the ordinal dependent variable in all ordered logistic regression models. The ordered logistic regression models were adjusted for the same list of covariates that were included in the four aforementioned logistic regression models. Covariates included in logistic regression models were selected as potential confounding factors based on their significance in univariable analyses or based on their biological plausibility.

All statistical tests were two-sided, and a *p*-value of < 0.05 (two-tailed) was considered to be statistically significant. Statistical analyses were performed using STATA software, version 16.1 (STATA, College Station, TX, USA).

## Figures and Tables

**Table 1:** Main clinical and biochemical characteristics of postmenopausal women with type 2 diabetes at baseline, stratified by worsening of glycaemic control at follow-up.

	Patients with no Worsening of Glycaemic Control at Follow-Up (*n* = 45)	Patients with Worsening of Glycaemic Control at Follow-Up (*n* = 16)	*p*-Value
Age (years)	70.9 ± 7.3	70.2 ± 9.0	0.738
BMI (kg/m^2^)	29.7 ± 5.5	29.5 ± 3.9	0.897
Diabetes duration (years)	10 (6–15)	10 (7–16)	0.810
Current smokers (%)	13.3	6.2	0.357
Systolic blood pressure (mmHg)	135 ± 14	139 ± 17	0.391
Diastolic blood pressure (mmHg)	77 ± 7	75 ± 10	0.413
Fasting glucose (mmol/L)	7.1 ± 1.7	7.2 ± 1.4	0.835
Haemoglobin A1c (mmol/mol Hb)	52 ± 9	53 ± 10	0.711
Haemoglobin A1c (%)	6.9 ± 0.8	7.0 ± 0.9	0.711
Proportion of patients with haemoglobin A1c (%) from 7% to 8% (53 to 64 mmol/mol Hb)	24.4	31.3	0.868
>8% (>64 mmol/mol Hb)	6.7	6.3	
Total cholesterol (mg/dL)	159 ± 31	166 ± 41	0.457
LDL-cholesterol (mg/dL)	79 ± 29	85 ± 34	0.512
HDL-cholesterol (mg/dL)	59 ± 14	58 ± 13	0.849
Triglycerides (mg/dL)	112 (72–150)	119 (92–167)	0.279
HOMA-IR score	2.3 (1.5–4.0)	3.3 (1.1–6.4)	0.422
AST (IU/L)	23 ± 7	25 ± 9	0.387
ALT (IU/L)	12 (10–16)	13 (10–18)	0.503
GGT (IU/L)	16 (13–28)	26 (16–36)	0.139
Creatinine (umol/L)	64 ± 13	66 ± 15	0.699
eGFR_CKD-EPI_ (ml/min/1.73 m^2^)	82 ± 14	81 ± 16	0.716
Hypertension (%)	73.3	87.5	0.318
Ischaemic heart disease (%)	13.3	6.3	0.664
Ischaemic stroke (%)	2.2	6.3	0.459
Diabetic retinopathy, any degree (%)	6.3	4.4	0.606
Metformin (%)	80.0	87.5	0.711
Sulfonylureas (%)	24.4	18.8	0.742
Pioglitazone (%)	2.2	0	0.738
DPP-4 inhibitors (%)	22.2	31.3	0.510
GLP-1 analogues (%)	8.9	12.5	0.648
SGLT-2 inhibitors (%)	11.1	0	0.313
Anti-platelets drugs (%)	42.2	31.3	0.557
Beta-blockers (%)	28.9	37.5	0.543
ACE-inhibitors/ARBs (%)	53.3	75.0	0.152
Calcium-channel blockers (%)	20.0	18.8	0.914
Diuretics (%)	31.1	37.5	0.758
Statins (%)	75.6	75.0	0.965
Fibroscan^®^-assessed LSM (kPa)	4.4 (3.6–5.6)	6.6 (5.4–8.6)	0.005
Patients with NAFLD and significant fibrosis ^§^ (%)	8.9	31.3	0.030
Patients with NAFLD and LSM ≥ 8.2% kPa (%)	6.7	25.0	0.044

Sample size, *n* = 61. Data are expressed as means ± SD, medians and IQRs (in parenthesis) or percentages. Differences between the two groups were tested by the Fisher’s exact test for categorical variables, the unpaired Student’s *t* test for normally distributed continuous variables and the Mann–Whitney test for non-normally distributed variables (i.e., diabetes duration, plasma triglycerides, HOMA-IR score, ALT, GGT and LSM). ^§^ Clinically significant fibrosis was defined by LSM ≥ 7 kPa on Fibroscan^®^. Hypertension was defined as blood pressure ≥ 140/90 mmHg and/or specific drug treatment. *Abbreviations*: ACE, angiotensin-converting-enzyme inhibitor; ARB, angiotensin II receptor blocker; ALT, alanine aminotransferase; AST, aspartate aminotransferase; BMI, body mass index; DPP-IV, dipeptidyl peptidase-IV; eGFR_CKD-EPI_, glomerular filtration rate estimated using the CKD-Epidemiology Collaboration equation; GGT, gamma-glutamyltransferase; GLP-1, glucagon-like peptide-1; HOMA-IR, homeostasis model assessment—insulin resistance; LSM, liver stiffness measurement; SGLT-2, sodium/glucose cotransporter-2.

**Table 2:** Main clinical and biochemical characteristics of postmenopausal women with type 2 diabetes at follow-up, stratified by severity of NAFLD at baseline.

	Patients with NAFLD Alone (*n* = 52)	Patients with NAFLD and Coexisting Significant Fibrosis (*n* = 9)	*p*-Value
Age (years)	75.7 ± 7.4	76.1 ± 8.9	0.874
Diabetes duration (years)	15 (11–21)	15 (11–18)	0.895
BMI (kg/m^2^)	29.5 ± 3.9	29.6 ± 4.3	0.961
Fasting glucose (mmol/L)	7.4 ± 1.9	8.8 ± 3.8	0.131
Haemoglobin A1c (mmol/mol Hb)	52 ± 9	68 ± 22	<0.001
Haemoglobin A1c (%)	6.9 ± 0.9	8.4 ± 2.1	<0.001
Proportion of patients with haemoglobin A1c (%) from 7% to 8% (53 to 64 mmol/mol Hb)	32.7	9.6	0.003
>8% (>64 mmol/mol Hb)	11.1	55.6	
Total cholesterol (mg/dL)	153 ± 35	141 ± 32	0.415
HDL-cholesterol (mg/dL)	54 ± 9	51 ± 22	0.603
Triglycerides (mg/dL)	112 (88–148)	79 (59–215)	0.488
AST (IU/L)	23 ± 9	20 ± 5	0.474
ALT (IU/L)	22 (16–27)	21 (14–25)	0.859
GGT (IU/L)	18 (11–60)	17 (10–41)	0.761
Creatinine (umol/L)	69 ± 24	74 ± 34	0.117
eGFR_CKD-EPI_ (ml/min/1.73 m^2^)	77.0 ± 19.0	67.8 ± 28.7	0.238
Hypertension (%)	78.9	88.9	0.484
Ischaemic heart disease (%)	9.6	11.1	0.633
Ischaemic stroke (%)	1.9	11.1	0.159
Diabetic retinopathy, any degree (%)	9.6	11.1	0.889
Insulin (%)	15.4	0	0.207
Metformin (%)	78.9	100.0	0.127
Sulfonylureas (%)	15.4	33.3	0.196
Pioglitazone (%)	1.9	0	0.675
DPP-4 inhibitors (%)	30.8	22.2	0.604
GLP-1 analogues (%)	23.1	33.3	0.509
SGLT-2 inhibitors (%)	23.1	33.3	0.509
Anti-platelets drugs (%)	32.7	44.4	0.493
Beta-blockers (%)	40.4	33.3	0.689
ACE-inhibitors/ARBs (%)	67.3	66.7	0.970
Calcium-channel blockers (%)	23.1	11.1	0.418
Diuretics (%)	32.7	44.4	0.493
Statins (%)	76.9	77.8	0.955

Sample size, *n* = 61. Data are expressed as means ± SD, medians and IQRs (in parenthesis) or percentages. Differences between the two groups were tested by the Fisher’s exact test for categorical variables, the unpaired Student’s *t* test for normally distributed continuous variables and the Mann–Whitney test for non-normally distributed variables. *Abbreviations*: ACE, angiotensin-converting enzyme; ARB, angiotensin II receptor blocker; ALT, alanine aminotransferase; AST, aspartate aminotransferase; BMI, body mass index; DPP-IV, dipeptidyl peptidase-IV; eGFR_CKD-EPI_, glomerular filtration rate estimated using the CKD-Epidemiology Collaboration equation; GGT, gamma-glutamyltransferase; GLP-1, glucagon-like peptide-1; SGLT-2, sodium/glucose cotransporter-2.

**Table 3:** Association between the severity of NAFLD at baseline and risk of developing worsening of glycaemic control at follow-up in postmenopausal women with type 2 diabetes.

Logistic Regression Models	Odds Ratios (95% CI)	*p*-Value
Unadjusted model		
NAFLD and clinically significant fibrosis ^§^	4.66 (1.07–20.3)	0.041
Adjusted model 1		
NAFLD and clinically significant fibrosis	4.72 (1.07–20.7)	0.040
Age (years)	0.98 (0.91–1.06)	0.678
BMI (kg/m^2^)	0.99 (0.88–1.12)	0.866
Adjusted model 2		
NAFLD and clinically significant fibrosis	4.70 (1.07–20.8)	0.041
Age (years)	0.99 (0.91–1.08)	0.724
BMI (kg/m^2^)	0.98 (0.87–1.11)	0.827
GLP-1 receptor agonist use	1.42 (0.21–10.1)	0.724
Adjusted model 3		
NAFLD and clinically significant fibrosis	4.99 (1.14–21.9)	0.033
Age (years)	0.96 (0.88–1.05)	0.376
BMI (kg/m^2^)	0.99 (0.87–1.12)	0.942
SGLT-2 inhibitor use	0.12 (0.10–3.07)	0.198
Adjusted model 4		
NAFLD and clinically significant fibrosis	4.11 (1.03–16.4)	0.045
Age (years)	0.99 (0.92–1.06)	0.744
BMI (kg/m^2^)	1.00 (0.89–1.11)	0.958
Pioglitazone use	1.21 (0.45–33.2)	0.907

Sample size, *n* = 61. Data are expressed as odds ratio and 95% confidence intervals (CI) as tested by logistic regression analyses. The presence of worsening of glycaemic control at follow-up (arbitrarily defined as HbA1c increase ≥ 0.5%) was the dependent variable in these logistic regression models. Covariates included in these regression models were recorded at baseline. ^§^ Clinically significant fibrosis was defined by LSM ≥ 7 kPa on Fibroscan^®^.

**Table 4:** Association between the severity of NAFLD at baseline and increasing levels of worsening of glycaemic control at follow-up in postmenopausal women with type 2 diabetes.

Ordered Logistic Regression Models	Odds Ratios (95% CI)	*p*-Value
Unadjusted model		
NAFLD and clinically significant fibrosis ^§^	6.16 (1.48–25.7)	0.013
Adjusted model 1		
NAFLD and clinically significant fibrosis	6.15 (1.47–25.9)	0.013
Age (years)	1.01 (0.95–1.08)	0.733
BMI (kg/m^2^)	0.97 (0.87–1.06)	0.462
Adjusted model 2		
NAFLD and clinically significant fibrosis	6.11 (1.46–26.6)	0.014
Age (years)	1.01 (0.95–1.09)	0.696
BMI (kg/m^2^)	0.96 (0.86–1.06)	0.431
GLP-1 receptor agonist use	1.53 (0.28–8.21)	0.621
Adjusted model 3		
NAFLD and clinically significant fibrosis	6.97 (1.62–30.0)	0.009
Age (years)	0.99 (0.92–1.06)	0.801
BMI (kg/m^2^)	0.96 (0.86–1.06)	0.385
SGLT-2 inhibitor use	0.14 (0.01–1.69)	0.123
Adjusted model 4		
NAFLD and clinically significant fibrosis	5.96 (1.42–25.1)	0.015
Age (years)	1.01 (0.95–1.08)	0.665
BMI (kg/m^2^)	0.96 (0.87–1.07)	0.481
Pioglitazone use	1.25 (0.40–31.0)	0.982

Sample size, *n* = 61. Data are expressed as odds ratio and 95% confidence intervals (CI) as tested by ordered logistic regression analyses. The presence of four increasing categories of worsening of glycaemic control at follow-up (i.e., arbitrarily defined as HbA1c increases ≤ 0.19%, from 0.20% to 0.49%, from 0.50% to 0.99% and ≥1%, respectively) was the ordinal dependent variable in all these models. All covariates included in these regression models were recorded at baseline. ^§^ Clinically significant fibrosis was defined by LSM ≥ 7 kPa on Fibroscan^®^.

## Data Availability

All data of the study are available in the manuscript.

## Supplementary Materials

The following supporting information can be downloaded at: https://www.mdpi.com/article/10.3390/ijms232012481/s1.

## References

[1] Riazi K., Azhari H., Charette J.H., Underwood F.E., King J.A., Afshar E.E., Swain M.G., Congly S.E., Kaplan G.G., Shaheen A.A. (2022). The prevalence and incidence of NAFLD worldwide: A systematic review and meta-analysis. Lancet Gastroenterol. Hepatol..

[2] Younossi Z.M., Golabi P., de Avila L., Paik J.M., Srishord M., Fukui N., Qiu Y., Burns L., Afendy A., Nader F. (2019). The global epidemiology of NAFLD and NASH in patients with type 2 diabetes: A systematic review and meta-analysis. J. Hepatol..

[3] Byrne C.D. (2022). Banting memorial lecture 2022: ‘Type 2 diabetes and nonalcoholic fatty liver disease: Partners in crime’. Diabet Med..

[4] Targher G., Corey K.E., Byrne C.D., Roden M. (2021). The complex link between NAFLD and type 2 diabetes mellitus—Mechanisms and treatments. Nat. Rev. Gastroenterol. Hepatol..

[5] Mantovani A., Turino T., Lando M.G., Gjini K., Byrne C.D., Zusi C., Ravaioli F., Colecchia A., Maffeis C., Salvagno G. (2020). Screening for non-alcoholic fatty liver disease using liver stiffness measurement and its association with chronic kidney disease and cardiovascular complications in patients with type 2 diabetes. Diabetes Metab..

[6] Stefan N., Cusi K. (2022). A global view of the interplay between non-alcoholic fatty liver disease and diabetes. Lancet Diabetes Endocrinol..

[7] Mantovani A., Petracca G., Beatrice G., Tilg H., Byrne C.D., Targher G. (2021). Non-alcoholic fatty liver disease and risk of incident diabetes mellitus: An updated meta-analysis of 501 022 adult individuals. Gut.

[8] Draznin B., Aroda V.R., Bakris G., Benson G., Brown F.M., Freeman R., Green J., Huang E., Isaacs D., American Diabetes Association Professional Practice Committee (2022). 4. Comprehensive Medical Evaluation and Assessment of Comorbidities: Standards of Medical Care in Diabetes-2022. Diabetes Care.

[9] Tilg H., Moschen A.R., Roden M. (2017). NAFLD and diabetes mellitus. Nat. Rev. Gastroenterol. Hepatol..

[10] Targher G., Byrne C.D. (2013). Clinical Review: Nonalcoholic fatty liver disease: A novel cardiometabolic risk factor for type 2 diabetes and its complications. J. Clin. Endocrinol. Metab..

[11] Ryysy L., Hakkinen A.M., Goto T., Vehkavaara S., Westerbacka J., Halavaara J., Yki-Jarvinen H. (2000). Hepatic fat content and insulin action on free fatty acids and glucose metabolism rather than insulin absorption are associated with insulin requirements during insulin therapy in type 2 diabetic patients. Diabetes.

[12] Afolabi B.I., Ibitoye B.O., Ikem R.T., Omisore A.D., Idowu B.M., Soyoye D.O. (2018). The Relationship Between Glycaemic Control and Non-Alcoholic Fatty Liver Disease in Nigerian Type 2 Diabetic Patients. J. Natl. Med. Assoc..

[13] Patel P.J., Hossain F., Horsfall L.U., Banh X., Hayward K.L., Williams S., Johnson T., Brown N.N., Saad N., Valery P.C. (2018). Controlled attenuation parameter in NAFLD identifies risk of suboptimal glycaemic and metabolic control. J. Diabetes Complicat..

[14] Targher G., Lonardo A., Byrne C.D. (2018). Nonalcoholic fatty liver disease and chronic vascular complications of diabetes mellitus. Nat. Rev. Endocrinol..

[15] Garcia-Compean D., Orsi E., Nishida T., Kumar R. (2022). Glycemic control, the unconsidered outcome in the treatment of nonalcoholic fatty liver disease. Ann. Hepatol..

[16] Wang B., Li M., Zhao Z., Wang S., Lu J., Chen Y., Xu M., Wang W., Ning G., Bi Y. (2020). Glycemic Measures and Development and Resolution of Nonalcoholic Fatty Liver Disease in Nondiabetic Individuals. J. Clin. Endocrinol. Metab..

[17] Sam S., Edelstein S.L., Arslanian S.A., Barengolts E., Buchanan T.A., Caprio S., Ehrmann D.A., Hannon T.S., Tjaden A.H., Kahn S.E. (2021). Baseline Predictors of Glycemic Worsening in Youth and Adults With Impaired Glucose Tolerance or Recently Diagnosed Type 2 Diabetes in the Restoring Insulin Secretion (RISE) Study. Diabetes Care.

[18] McGovern A.P., Dennis J.M., Shields B.M., Hattersley A.T., Pearson E.R., Jones A.G., Consortium M. (2019). What to do with diabetes therapies when HbA1c lowering is inadequate: Add, switch, or continue? A MASTERMIND study. BMC Med..

[19] Alexopoulos A.S., Crowley M.J., Wang Y., Moylan C.A., Guy C.D., Henao R., Piercy D.L., Seymour K.A., Sudan R., Portenier D.D. (2021). Glycemic Control Predicts Severity of Hepatocyte Ballooning and Hepatic Fibrosis in Nonalcoholic Fatty Liver Disease. Hepatology.

[20] Hamaguchi E., Takamura T., Sakurai M., Mizukoshi E., Zen Y., Takeshita Y., Kurita S., Arai K., Yamashita T., Sasaki M. (2010). Histological course of nonalcoholic fatty liver disease in Japanese patients: Tight glycemic control, rather than weight reduction, ameliorates liver fibrosis. Diabetes Care.

[21] Tanaka K., Takahashi H., Hyogo H., Ono M., Oza N., Kitajima Y., Kawanaka M., Chayama K., Saibara T., Anzai K. (2019). Epidemiological survey of hemoglobin A1c and liver fibrosis in a general population with non-alcoholic fatty liver disease. Hepatol. Res..

[22] Mantovani A., Valenti L. (2021). A call to action for fatty liver disease. Liver Int.

[23] Targher G., Tilg H., Byrne C.D. (2021). Non-alcoholic fatty liver disease: A multisystem disease requiring a multidisciplinary and holistic approach. Lancet Gastroenterol. Hepatol..

[24] Byrne C.D., Targher G. (2022). Non-alcoholic fatty liver disease is a risk factor for cardiovascular and cardiac diseases: Further evidence that a holistic approach to treatment is needed. Gut.

[25] Mantovani A., Byrne C.D., Targher G. (2022). Efficacy of peroxisome proliferator-activated receptor agonists, glucagon-like peptide-1 receptor agonists, or sodium-glucose cotransporter-2 inhibitors for treatment of non-alcoholic fatty liver disease: A systematic review. Lancet Gastroenterol. Hepatol..

[26] Petroni M.L., Brodosi L., Bugianesi E., Marchesini G. (2021). Management of non-alcoholic fatty liver disease. BMJ.

[27] Mantovani A., Petracca G., Beatrice G., Csermely A., Lonardo A., Targher G. (2021). Glucagon-Like Peptide-1 Receptor Agonists for Treatment of Nonalcoholic Fatty Liver Disease and Nonalcoholic Steatohepatitis: An Updated Meta-Analysis of Randomized Controlled Trials. Metabolites.

[28] Mantovani A., Petracca G., Csermely A., Beatrice G., Targher G. (2020). Sodium-Glucose Cotransporter-2 Inhibitors for Treatment of Nonalcoholic Fatty Liver Disease: A Meta-Analysis of Randomized Controlled Trials. Metabolites.

[29] Davies M.J., Aroda V.R., Collins B.S., Gabbay R.A., Green J., Maruthur N.M., Rosas S.E., Del Prato S., Mathieu C., Mingrone G. (2022). Management of Hyperglycemia in Type 2 Diabetes, 2022. A Consensus Report by the American Diabetes Association (ADA) and the European Association for the Study of Diabetes (EASD). Diabetes Care.

[30] Lonardo A., Nascimbeni F., Ballestri S., Fairweather D., Win S., Than T.A., Abdelmalek M.F., Suzuki A. (2019). Sex Differences in Nonalcoholic Fatty Liver Disease: State of the Art and Identification of Research Gaps. Hepatology.

[31] Byrne C.D., Patel J., Scorletti E., Targher G. (2018). Tests for diagnosing and monitoring non-alcoholic fatty liver disease in adults. BMJ.

[32] Gawrieh S., Knoedler D.M., Saeian K., Wallace J.R., Komorowski R.A. (2011). Effects of interventions on intra- and interobserver agreement on interpretation of nonalcoholic fatty liver disease histology. Ann Diagn Pathol.

[33] Cengiz M., Senturk S., Cetin B., Bayrak A.H., Bilek S.U. (2014). Sonographic assessment of fatty liver: Intraobserver and interobserver variability. Int. J. Clin. Exp. Med..

[34] Liu J., Ma Y., Han P., Wang J., Liu Y.G., Shi R.F., Li J. (2022). Hepatic steatosis leads to overestimation of liver stiffness measurement in both chronic hepatitis B and metabolic-associated fatty liver disease patients. Clin. Res. Hepatol. Gastroenterol..

[35] Mantovani A., Sani E., Fassio A., Colecchia A., Viapiana O., Gatti D., Idolazzi L., Rossini M., Salvagno G., Lippi G. (2019). Association between non-alcoholic fatty liver disease and bone turnover biomarkers in post-menopausal women with type 2 diabetes. Diabetes Metab..

[36] Levey A.S., Stevens L.A., Schmid C.H., Zhang Y.L., Castro A.F., Feldman H.I., Kusek J.W., Eggers P., Van Lente F., Greene T. (2009). A new equation to estimate glomerular filtration rate. Ann. Intern. Med..

[37] Wong V.W., Vergniol J., Wong G.L., Foucher J., Chan H.L., Le Bail B., Choi P.C., Kowo M., Chan A.W., Merrouche W. (2010). Diagnosis of fibrosis and cirrhosis using liver stiffness measurement in nonalcoholic fatty liver disease. Hepatology.

