# Supplementary material for: Long-Term Adverse Effect of Liver Stiffness on Glycaemic Control in Type 2 Diabetic Patients with Nonalcoholic Fatty Liver Disease: A Pilot Study
Source: Int J Mol Sci. 2022 Oct 18;23(20):12481. doi: 10.3390/ijms232012481 (PMC9604384; doi:10.3390/ijms232012481)
Supplement: Supplementary file 1 [file ijms-23-12481-s001.zip › ijms-1922208-supplementary.pdf]

SUPPLEMENTARY MATERIALS

Supplementary Table S1. Association between the severity of NAFLD at baseline and risk of developing worsening of glycaemic control at follow-up in post-menopausal women with type 2 diabetes.

**Logistic regression models**	**Odds Ratios (95% CIs)**	***P* values**
**Adjusted model 1**		
NAFLD and clinically significant fibrosis§	4.91 (1.05-22.9)	**0.043**
Age (years)	0.98 (0.91-1.06)	0.667
BMI (kg/m^2)	0.99 (0.88-1.12)	0.867
HbA1c (%)	0.93 (0.43-1.99)	0.854
**Adjusted model 2**		
NAFLD and clinically significant fibrosis	4.97 (1.06-23.4)	**0.043**
Age (years)	0.98 (0.91-1.07)	0.711
BMI (kg/m^2)	0.99 (0.87-1.11)	0.822
HbA1c (%)	0.91 (0.41-1.99)	0.804
GLP-1 receptor agonist use	1.49 (0.21-10.7)	0.694
**Adjusted model 3**		
NAFLD and clinically significant fibrosis	4.77 (1.06-21.6)	**0.042**
Age (years)	0.96 (0.89-1.05)	0.382
BMI (kg/m^2)	0.99 (0.88-1.11)	0.940
HbA1c (%)	1.02 (0.51-2.38)	0.964
SGLT-2 inhibitor use	0.12 (0.10-3.05)	0.199
**Adjusted model 4**		
NAFLD and clinically significant fibrosis	4.70 (1.00-22.1)	**0.050**
Age (years)	0.99 (0.92-1.06)	0.712
BMI (kg/m^2)	1.00 (0.89-1.11)	0.954
HbA1c (%)	0.99 (0.49-2.01)	0.977
Pioglitazone use	1.21 (0.40-36.5)	0.977

Sample size, *n*=61. Data are expressed as odds ratio and 95% confidence intervals (CI) as tested by logistic regression analyses. The presence of worsening of glycaemic control at follow-up (defined as HbA1c increase ≥0.5%) was the dependent variable in these logistic regression models. All covariates included in these regression models were recorded at baseline. §Clinically significant fibrosis was defined by LSM ≥7 kPa on Fibroscan®. For the sake of clarity, significant *p*-values have been highlighted in bold.

Supplementary Table S2. Ordered logistic regression analyses - Association between the severity of NAFLD at baseline and increasing levels of worsening of glycaemic control at follow-up in post-menopausal women with type 2 diabetes.

Ordered logistic regression models	Odds Ratios (95% CIs)	P values
**Adjusted model 1**		
NAFLD and clinically significant fibrosis§	6.49 (1.47-28.6)	**0.014**
Age (years)	1.01 (0.95-1.08)	0.760
BMI (kg/m^2)	0.96 (0.87-1.07)	0.468
HbA1c (%)	0.91 (0.49-1.69)	0.765
**Adjusted model 2**		
NAFLD and clinically significant fibrosis	6.51 (1.48-28.7)	**0.013**
Age (years)	1.01 (0.95-1.08)	0.725
BMI (kg/m^2)	0.96 (0.87-1.06)	0.434
HbA1c (%)	0.89 (0.47-1.68)	0.719
GLP-1 receptor agonist use	1.58 (0.29-8.54)	0.593
**Adjusted model 3**		
NAFLD and clinically significant fibrosis	7.10 (1.58-31.9)	**0.011**
Age (years)	0.99 (0.92-1.06)	0.796
BMI (kg/m^2)	0.96 (0.86-1.06)	0.388
HbA1c (%)	0.97 (0.52-1.80)	0.915
SGLT-2 inhibitor use	0.14 (0.01-1.69)	0.126
**Adjusted model 4**		
NAFLD and clinically significant fibrosis	6.11 (1.39-26.9)	**0.017**
Age (years)	1.01 (0.95-1.08)	0.680
BMI (kg/m^2)	0.96 (0.87-1.07)	0.483
HbA1c (%)	0.96 (0.51-1.79)	0.894
Pioglitazone use	1.25 (0.40-31.0)	0.972

Sample size, *n*=61. Data are expressed as odds ratio and 95% confidence intervals (CI) as tested by ordered logistic regression analyses. The presence of four increasing categories of worsening of glycaemic control at follow-up (i.e., arbitrarily defined as HbA1c increases ≤0.19%, from 0.20% to 0.49%, from 0.50% to 0.99%, and ≥1%, respectively) was the ordinal dependent variable in all these models. All covariates included in these regression models were recorded at baseline. §Clinically significant fibrosis was defined by LSM ≥7 kPa on Fibroscan®. For the sake of clarity, significant *p*-values have been highlighted in bold.
